# MicroSyn: A user friendly tool for detection of microsynteny in a gene family

**DOI:** 10.1186/1471-2105-12-79

**Published:** 2011-03-18

**Authors:** Bin Cai, Xiaohan Yang, Gerald A Tuskan, Zong-Ming Cheng

**Affiliations:** 1 College of Horticulture, Nanjing Agricultural University, Nanjing, China; 2 Department of Plant Sciences, University of Tennessee, Knoxville, TN, USA; 3 Biosciences Division, Oak Ridge National Laboratory, Oak Ridge, TN, USA; 4 BioEnergy Science Center, Oak Ridge National Laboratory, Oak Ridge, TN, USA

## Abstract

**Background:**

The traditional phylogeny analysis within gene family is mainly based on DNA or amino acid sequence homologies. However, these phylogenetic tree analyses are not suitable for those "non-traditional" gene families like microRNA with very short sequences. For the normal protein-coding gene families, low bootstrap values are frequently encountered in some nodes, suggesting low confidence or likely inappropriateness of placement of those members in those nodes.

**Results:**

We introduce MicroSyn software as a means of detecting microsynteny in adjacent genomic regions surrounding genes in gene families. MicroSyn searches for conserved, flanking colinear homologous gene pairs between two genomic fragments to determine the relationship between two members in a gene family. The colinearity of homologous pairs is controlled by a statistical distance function. As a result, gene duplication history can be inferred from the output independent of gene sequences. MicroSyn was designed for both experienced and non-expert users with a user-friendly graphical-user interface. MicroSyn is available from: http://fcsb.njau.edu.cn/microsyn/.

**Conclusions:**

Case studies of the microRNA167 genes in plants and Xyloglucan ndotransglycosylase/Hydrolase family in *Populus trichocarpa *were presented to show the utility of the software. The easy using of MicroSyn in these examples suggests that the software is an additional valuable means to address the problem intrinsic in the computational methods and sequence qualities themselves in gene family analysis.

## Background

Over the past ~ 200 million years, flowering plant (angiosperm) genomes have undergone multiple whole-genome duplications (WGDs), followed by chromosomal rearrangement, gene shuffling and gene loss after each duplication, as well as subsequent inversions, translocations and tandem duplications [1]. In addition, among short DNA segments mobile elements like retrotransposons have caused additional rearrangements and partial or complete gene duplications. As a result, extant angiosperms vary more than 1000-fold in genome sizes and nearly 50-fold in chromosome numbers [2]. Such multilevel rearrangements, accompanied by simultaneous gene loss and tandem duplications, make it very challenging to understand genome evolutions, to infer paleo-polyploidy and to determine the orthology and paralogy among and within plant species.

Fortunately, with the availability of several fully sequenced plant genomes, studies of genome evolution and organization become possible in part by detecting syntenies and colinearities or clustering and ordering of neighboring matching gene pairs [3]. Typically, synteny and colinearity have been identified by searching for statistically supportive conservation between (pairwise) or among (multiway) species [4]. Recent studies on conserved syntenic regions across various plant species at the whole-genome level have provided valuable insights into the evolution and organization of whole genomes and the homology in several plant species [3,5,6]. Microsynteny (small-scale of synteny) has been investigated across several plant species using whole-genome sequences or selected discrete sequences to infer shared ancestry [7,8].

Inferring the evolutionary history of a gene family and related chromosome segments is one of the main tasks in studying biological evolution. The traditional phylogenetic principles and methods are based on amino acid or nucleotide sequence to assess homology, determine orthology and paralogy, and deduce relationships within gene families and/or reconstruct gene evolutionary events. Several approaches have been developed including parsimony, phonetic and maximum likelihood; each has drawbacks, especially when the sequences of the interested genes are truncated, contain gaps or are low quality [9]. Since traces from past evolution events for a gene family can often be detected from the local genome organization [10], we may be able to take advantage of microsynteny to better infer gene family evolution.

The identification of synteny, whether macro- or micro-synteny, can be laborious and error-prone, and is usually performed manually with some basic tools such as BLAST [11]. Some software packages have been developed to facilitate such searches. ADhoRe was developed to find synteny, particularly for larger genomic regions [12]. An automatic microsynteny analysis pipeline and a synteny database have been developed for animals [13]. However, these tools and pipeline are case-specific or for specialists with extensive training and experiences. Their limited user interface makes themselves difficult to be used by users without specific training; a graphical user interface (GUI) is not available in current microsynteny analysis tools.

We have developed a tool, called MicroSyn, to semi-automatically detect microsynteny within an individual gene family. In particular, MicroSyn was designed for both experienced and non-expert users. To demonstrate the utility and use of MicroSyn, we presented a case study of the evolution of miR167 gene family in *Arabidopsis thaliana *(Arabidopsis), *Populus trichocarpa *(*Populus*), *Vitis vinifera *(grape) and *Oryza sativa *(rice). We demonstrate how MicroSyn can be used to overcome problems with the reconstruction of phylogenetic relationships from short nucleic acid sequences in that the quality of the sequence alignments rapidly declines when the pair-wise sequence identity falls below 50 or 60%. The precursor sequences of homologous microRNAs (miRNAs) are often below this threshold. Additionally, we also conducted a case study to confirm the utility of MicroSyn in verifying the phylogenetic relationships of genes in a traditional protein-coding gene family, using Xyloglucan Endotransglycosylase/Hydrolase (XTH) genes in *Populus *as a test case.

## Implementation

**Figure 1:**
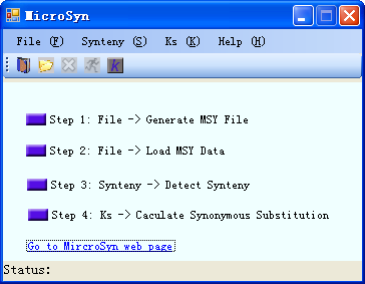
**A screenshot of the MicroSyn main window**. Screenshot of MicroSyn main window, which is a GUI interface, facilitate users to operate through menu or toolbars.

The software was written in C# language. This tool has been successfully tested on Windows platforms. In designing the software, user's interface was considered to be an important feature. To this end, a GUI of the program is provided (Figure 1); all operations can be done just by clicking the computer mouse. Here we describe the detailed algorithms of MicroSyn.

### Algorithm

#### Searching strategy

**Figure 2:**
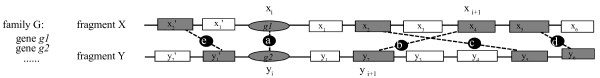
**Diagram of two genomic segments containing the genes to be analyzed and their flanking genes**. Every box stands for a gene. Boxes *g1 *and *g2 *denote members in a family "G" and "fragment X" represents the genomic fragment that contains gene *g1 *and its neighboring genes and "fragment Y" is the genomic fragment that contains *g2 *and its neighboring genes. The gray boxes connected by dashed lines represent pairs of homologous genes (a, b, c, d, e) between fragment X and Y. When calculate distance between two adjacent gene pairs, we consider one homologous pair with coordinate (*x_i _*, *y_i_*) and another pair with (*x*_*i*+1 _, *y*_*i*+1_) on fragments.

To study the relationship between two members in a gene family, the DNA sequence containing their neighboring genes are considered. As shown in Figure 2, gene *g1 *and *g2 *are the two members of gene family G and the genomic fragment X contains gene *g1 *and its neighboring genes; the genomic fragment Y contains *g2 *and its neighboring genes. The homologous pairs are represented as two gray rectangles connected with dashed line and there are five homologous pairs (a, b, c, d, e) between genomic fragments X and Y. To study the relationship between *g1 *and *g2*, MicroSyn uses the homologous relationship between genes on fragments X and Y. The algorithm takes the gene pair (gene *g1 *and *g2*) as a starting point to search neighboring homologous pairs. In the process of searching, the software always determines the best adjacent homologous gene pair which remains the best colinearity on the genomic fragments. For example, for pair "a", there are two pairs (b and c) to be selected (Figure 2). To determine the best adjacent gene pair, we adopt the distance (*d_i_*) function that was introduced in ADhoRe [14]. Consider one homologous pair with coordinate (*x_i _, y_i_*) and another pair with (*x*_*i*+1 _, *y*_*i*+1_). The *d_i _*between two adjacent homology gene pairs (*x_i _*, *y_i_*) and (*x*_*i*+1 _, *y*_*i*+1_) is given by:

The distance controls the extent of the colinearity for a series of homologous pairs between two fragments. In this way, the homologous pairs that are located in minimum distance are recorded and then the search continues to the next gene pair. According to this criterion, pair "b" is recorded and taken as the new point for searching the adjacent homologous pair. Finally, all the fitted pairs are stored into one cluster that contains gene pairs a, b, d and e in Figure 2. The cluster represents the conserved region between two genomic fragments that have evolution relationship. The searching process is mainly controlled by two parameters: the maximum distance, *max*(|*x*_*i*+1 _- *x_i_*|, |*y*_*i*+1 _- *y_i_*|), between two adjacent genes in each fragment and the actual value of *d_i _*between two adjacent homologous pairs.

#### Statistical validation of conserved genomic fragment

To discard a negative cluster that is likely generated by chance, a statistical assessment was developed. Consider two fragments with number of *m *and *n *genes, respectively, and the number of homologous gene pairs represented by *l*. The probability of finding a homologous pair in the two fragments is given by:

Next, considering the detected cluster containing homologous pairs and a searching range of  gene pairs from two fragments, within the context of a binomial distribution, the gene pair is observed by chance with the probability:

Thus, when MicroSyn starts searching from a specific gene pair, it detects homologous pairs in an area of , where *k *equals the number of homologous gene pairs in the cluster. Thus, the final probability to find the cluster containing homologous gene pairs by chance is given by:

In other words, the value of *p_c _*is determined by both the number of homologous gene pairs in a given detected cluster and the total number of genes in two genomic fragments. So, if *p_c _*of a cluster exceeds a threshold value, the cluster is considered to be negative and should be discarded.

## Result

This section describes the pipeline of the tool and its application to two examples of gene families in plants.

### Pipeline

#### Step 1: Generate MSY file

**Figure 3:**
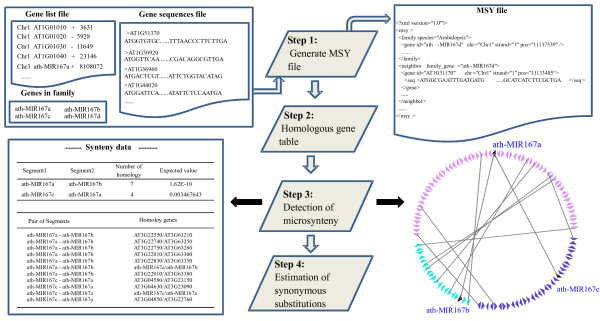
**Pipeline of the MicroSyn**. In the gene list file, one line contains chromosome or fragment name, gene accession number, the '+' or '-' sign indicating the orientation of the gene on strand, and the absolute or relative position. Gene sequence file contains corresponding gene's CDS in FASTA format. These two files and genes in a family are merged into a file, called MSY file here. After loading the MSY file, MicroSyn then initially creates a homologous relationship among all genes on fragments and a table consisting of all pairs of homologous genes is obtained. Next, MicroSyn begins detecting microsynteny between fragments that contain the members in a gene family. Finally, the software also estimates the level of Ks of paralogs between two fragments, using the flanking gene sequences.

The conceptual pipeline is showed in Figure 3. To detect microsynteny between genes in a family, MicroSyn requires: 1) names of genes in the family of interest, 2) a data set representing genomic fragments (refer to as gene list) containing the genes in the family and their flanking gene names, their genomic positions and orientations and 3) all predicted gene coding sequences (CDSs). Users are recommended to obtain gene families from some online databases, such as Phytozome http://www.phytozome.net/, PLAZA [15], GreenPhylDB [16], etc. The gene list can be generated based on user's experimental data or it can be downloaded from a pre-calculated gene list at our web site http://fcsb.njau.edu.cn/microsyn/. These gene lists were extracted from whole-genomic GFF file of sequenced species. The count of flanking genes can be set and all above information is merged into a MSY file that can be loaded into the software when a user needs. The MSY file is in Extensible Markup Language (XML) format; one can edit contents of the data set in the MSY file using a text editor if some minor changes need to be made.

#### Step 2: Homologous gene table

The CDSs in MSY file are first translated into corresponding protein sequences by MicroSyn. For gene protein sequences from two gene fragments, for which microsynteny is to be detected, an all-against-all sequence similarity search is initially performed using BLASTP. Homologous genes are determined if two protein sequences have ≥30% sequence identity over an aligned region of ≥100 amino acids. After parsing the sequence similarity search results, a table consisting of all pairs of homologous genes is obtained. For recently tandem-duplicated genes, only the member at the first position is included as a representative for tandem homologous genes.

#### Step 3: Detection of microsynteny

The homologous gene pairs between two genomic fragments are searched iteratively according to a distance function that controls the colinearity of homologous pairs (see details in algorithm). The selected gene pairs which satisfy the criterion are placed in a cluster which contains the information on microsynteny of two gene fragments. The result of MicroSyn is presented visually and saved in an output file. The graphic file provides an intuitive view of microsynteny that can be saved as an image file. The other information is placed in text format in a main output window (Figure 3).

#### Step 4: Estimation of synonymous substitutions

The peptide alignments, resulting from BLASTP, of each pair of conserved flanking genes surrounding the target gene(s) are taken as a guide to extract highly quality aligned coding sequence, excluding gaps. The level of synonymous substitution (Ks) for the selected nucleotide sequences is calculated using the method by Nei and Gojobori [17]. The mean Ks value is calculated for all pairs of protein-coding genes between each of genomic fragments.

### Case Studies

In this study, we demonstrate the utility of MicroSyn by applying it to exploration of two gene families in plants.

#### Sequence resource for case studies

The precursor miR167s sequences of Arabidopsis, *Populus*, grape and rice were downloaded from the miRNA Registry version 13 http://microrna.sanger.ac.uk/. The coordinates of miR167s on Arabidopsis and rice were also downloaded from the miRNA Registry. For *Populus *and grape miR167s, the coordinates on newly released genome annotation is not available; we calculated the coordinates of miR167s by aligning each gene against the whole-genome assembly. The GFF and complete CDSs of plant species were obtained from public domains. The Arabidopsis CDSs and GFF were downloaded from TAIR release 9 ftp://ftp.arabidopsis.org/home/tair/Genes/TAIR9_genome_release/. The *Populus *CDSs and GFF were downloaded from ftp://ftp.jgi-psf.org/pub/JGI_data/phytozome/v5.0/Ptrichocarpa/annotation/. The grape CDSs and GFF were downloaded from http://www.genoscope.cns.fr/externe/Download/Projets/Projet_ML/data/12X/annotation/. The rice CDSs and GFF were downloaded from ftp://ftp.plantbiology.msu.edu/pub/data/Eukaryotic_Projects/o_sativa/annotation_dbs/pseudomolecules/version_6.0/all.dir/.

A total of 39 XTH genes in *Populus *was identified by using the HMMER3 http://hmmer.janelia.org/ and the PF06955 Hidden Markov Model http://pfam.wustl.edu, with the gathering cutoff option (--cut_ga). Multiple protein sequences then were aligned using CLUSTAL W2 [18] with default parameters. The neighbor jointing (NJ) phylogenetic tree and maximum likelihood (ML) tree were constructed using PHYLIP [19] with default options. Resampling was performed using bootstrapping with replicates of 100.

#### Evolution of miR167 gene family in plants

We applied MicroSyn to study the miR167 gene family in Arabidopsis, *Populus*, grape and rice, four of the plant species with available whole-genome sequence. We obtained all 27 precursor miR167s sequences of Arabidopsis, *Populus*, grape and rice from the miRNA database http://microrna.sanger.ac.uk/. The information of miR167s in these four species is listed in Table 1 and was used as input to the gene list that is required by MicroSyn. The microsynteny relationship of a gene family calculated by MicroSyn is represented both in graph and text. Then the microsynteny information is transformed to the order of duplication events using "degree of conservation of microsynteny", a principle inspired by a previous paper [10]. This means that if the microsynteny between two members of a gene family is more significant, these two members evolved from a duplication event more recently.

**Table 1:** Basic information on the miR167 families in Arabidopsis, *Populus*, grape and rice

Species	Chromosome	Position	Strand	ID
Arabidopsis	Chr1	11137539	+	ath-miR167d
	Chr3	1306622	-	ath-miR167c
	Chr3	8108072	+	ath-miR167a
	Chr3	23406168	+	ath-miR167b
*Populus*	Chr2	3062198	-	ptc-miR167a
	Chr2	3064788	-	ptc-miR167b
	Chr5	22458162	+	ptc-miR167c
	Chr5	22447920	+	ptc-miR167d
	Chr5	3575879	+	ptc-miR167e
	Chr10	10015916	-	ptc-miR167f
	Chr8	10561904	+	ptc-miR167g
	Chr13	2408396	+	ptc-miR167h
grape	Chr1	1618504	+	vvi-miR167a
	Chr14	7137388	+	vvi-miR167b
	Chr Unk	7495686	+	vvi-miR167c
	Chr Unk	7490493	+	vvi-miR167d
	Chr5	5845385	+	vvi-miR167e
rice	Chr1	32685024	-	osa-miR167j
	Chr2	3742238	-	osa-miR167e
	Chr3	3346678	+	osa-miR167g
	Chr3	30539817	-	osa-miR167b
	Chr3	33123489	+	osa-miR167c
	Chr6	27673751	+	osa-miR167i
	Chr7	4165296	-	osa-miR167d
	Chr10	14651810	-	osa-miR167f
	Chr12	25443203	+	osa-miR167a
	Chr12	25447013	+	osa-miR167h

##### Relationship and evolution of miR167s within Arabidopsis, Populus, grape and rice

**Figure 4:**
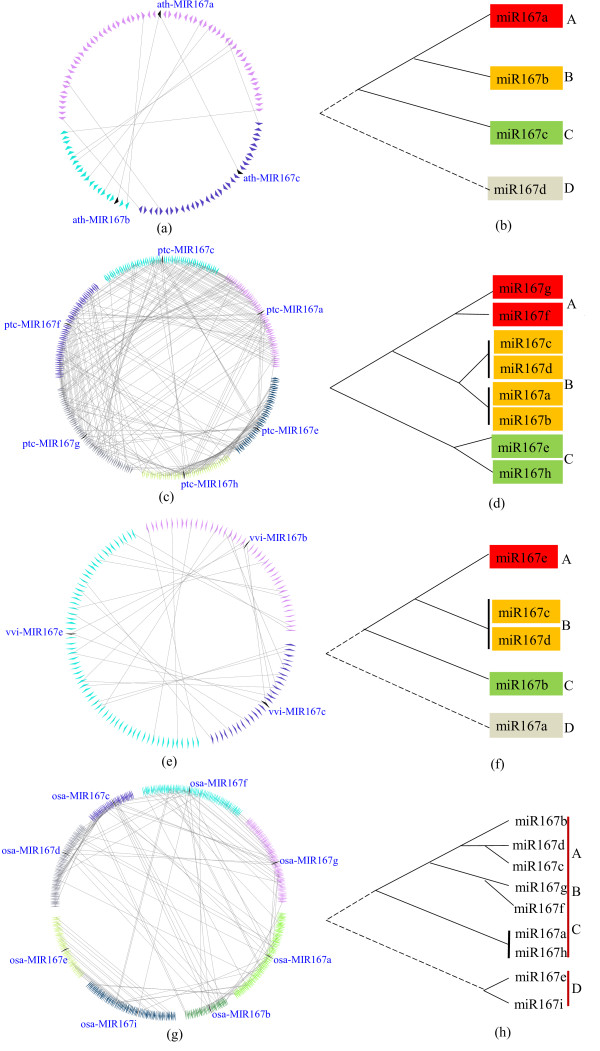
**Microsynteny related to miR167 families in a) Arabidopsis; c) *Populus*; e) grape; g) rice and reconstruction of the miRNA167 families evolution in b) Arabidopsis; d) *Populus*; f) grape; h) rice**. a, c, e, g: The genomic fragment is represented by a series of triangles that represent a gene in a family and its flanking genes. The genes in the same fragment show the same color except the gene in a family which is shaded by black triangle. The triangle also indicates gene's orientation on strands. The homologous genes on two fragments are connected by a gray line. b, d, f, h: These evolution relationship were generated to demonstrate the order of duplication events for the miR167 families in respective species. Black vertical line indicates tandem duplication. The orthologous miRNAs are classified into four orthologous groups designated as A, B, C and D, each of which is represented by the same color. Group D miRNAs are most ancient and the colinearity found between the group D and other groups are not significant, therefore, the group D is represented by a dashed line. In rice, miR167s out of the group D are designated as group A/B/C.

We first analyzed the relationship of miR167 genes within each species to infer gene duplication events. There are four miR167 genes in Arabidopsis. miR167a and miR167b have conserved neighboring regions; with less conserved colinear genes surrounding miR167a and miR167c and no conserved colinear genes between miR167b and miR167c (Figure 4a). MicroSyn did not detect microsynteny between miR167d and other family members. Based on the predicted syntenic relationships, Arabidopsis miR167a and miR167b appear to have evolved from a single duplication event, while miR167c existed prior to this duplication event and miR167d is an ancient gene that has no detected linkage with other miR167 genes (Figure 4b).

Eight miR167 genes were identified in *Populus*. The tandemly duplicated miR167a and miR167b share substantial colinear region with the tandemly duplicated miR167c and miR167d (Figure 4c). miR167f and miR167g share conserved synteny and microsynteny was detected between miR167e and miR167h. According to the extent of conservation within this gene family, miR167f/miR167g and miR167a/miR167b/miR167c/miR167d arose from a duplication event, and the common ancestor of miR167f/miR167g and miR167a/miR167b/miR167c/miR167d, might be evolved from duplication after the ancestor of miR167e/miR167h appeared (Figure 4d).

In grape, there are five members in the miR167 family. The microsynteny between the miR167c and miR167e is extensive, followed by the miR167b and miR167e and then miR167b and miR167c (Figure 4e). Interestingly, the region around miR167a lacks detectable colinear relationship to all other miR167 genes in grape. The predicted syntenic relationships suggest that miR167c/miR167d and miR167e arose from a recent duplication event, and that the miR167b and the ancestor of miR167c/miR167d and miR167e evolved from an ancient duplication (Figure 4f). The region of miR167a lacks significant colinear relations to all other miR167 genes; therefore, miR167a is placed close to miR167b as an outlier (Figure 4f).

Ten miR167 genes were identified in rice. The miR167a and miR167h are tandem genes located on chromosome 12. Microsynteny was detected between 1) miR167c and miR167d, 2) miR167f and miR167g and 3) miR167e and miR167i (Figure 4g). These gene pairs all have conservative regions with miR167a. For miR167j, no conserved regions were observed. In rice, three pairs (miR167c/miR167d, miR167f/miR167g and miR167e/miR167i) appear to have evolved from a recent duplication event as these gene pairs all have a conservative region with miR167a (Figure 4h).

##### Relationship and evolution of miR167s in eudicots

**Figure 5:**
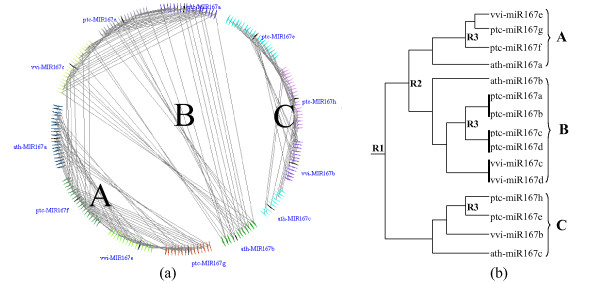
**Microsynteny related to miR167 family across three eudicots species (Arabidopsis, *Populus *and grape) and the duplication events of miR167 family in these species**. a: The colinear homologous genes are linked by gray lines. The homologous relationships are obviously clustered in three groups designed A, B and C in Arabidopsis, *Populus *and grape. Use: ptc for *Populus*, ath for Arabidopsis, vvi for grape. b: The evolution relationship was generated to demonstrate the order of duplication events for the miR167 family in eudicots species. The gene duplication events are designated as R1, R2 and R3.

To clarify the relationship of miR167s across eudicots, the microsynteny of miR167 genes across Arabidopsis, *Populus *and grape was then examined. Four orthologous groups were predicted and are designated as A, B, C and D (Figure 4; Figure 5). miR167a in Arabidopsis, miR167f and miR167g in *Populus*, and miR167e in grape have a conserved colinearity, and are classified into the group "A". miR167b in Arabidopsis, miR167a/miR167b/miR167c/miR167d in *Populus*, and miR167c/miR167d in grape are identified as group "B". miR167c in Arabidopsis, miR167e/miR167h in *Populus*, and miR167b in grape are grouped as group "C". miR167d in Arabidopsis, and miR167a in grape are grouped as group "D" (Figure 4).

It is thought that miRNA families evolved from a process of genome-wide duplication, tandem duplication and segmental duplication accompanied by post-duplication diversification, similar to the evolution process of protein-coding gene families [10]. Here we found that the miR167 homologs appear to have arisen via gene duplication events, which are designated as R1, R2 and R3 in this study (Figure 5). Group D appears to be most ancient and few traces of colinearity can be found between it and other groups, indicating either that it evolved via a small duplication event that did not involve surrounding genes, or that microsynteny is too ancient to be detected, or that it occurred via complete transposition and loss of its progenitor.

From the orthologous group model across three lineages and the synteny relationship within species, we presume that miR167 duplication of group C and the common ancestor of group A and B occurred at R1 and group A and B evolved at R2. The R3 duplication is only founded in *Populus*. The paralogs evolved from R3 duplication are located on different chromosomes (e.g., miR167f on chromosome 10, miR167g on chromosome 8), indicating that they likely arose from a whole-genome duplication event. In addition, the levels of Ks in duplicated pairs formed by R3 duplication event between mi167s range from 0.29 to 0.33 (Table 2). Based on the Ks values of R3 duplication event using substitutions/synonymous site/year (1.5 × 10^-8^) observed in Arabidopsis, we obtained that the R3 occurred approximate 10 million years ago (MYA). Tuskan et al. [20] proposed that recurrent contributions of *Populus *gametes to multiple generations could slow the molecular clock in *Populus*, ticking at only one-sixth the estimated rate in *Populus *comparing that in Arabidopsis. Using this one-sixth as correction factor, the date of R3 would have actually occurred around 60 MYA, or approximately matching to the fossil record where the salicoid duplication occurred right before separation of *Populus *and willow in approximately 65 MYA [20]. So, we conclude that R3 corresponds to the salicoid duplication ρ in *Populus*. The paralogs evolved from the R2 have the high level of Ks, e.g., Ks = 1.66-1.79 in *Populus *and Ks = 1.83 in Arabidopsis. The orthologous relationship between Arabidopsis and *Populus *miR167s indicates that R2 duplication occurred prior to the split of Arabidopsis and *Populus*. For the grape R2 duplication, the value of Ks between homologous fragments in our study reflects some different trajectories than those in Arabidopsis and *Populus*. The Ks between group A (miR167e) and group B (miR167c) is 1.33 and the Ks between group A (miR167e) and group C (miR167b) is 1.32 (Table 2). There is then a paradox; that is, miR167e and miR167c and miR167e and miR167b have Ks = 1.3 while miR167c and miR167b have a Ks = 2.03. The Ks related to miR167e seems to be "abnormal" compared to the Ks values of paralogous gene pairs. However, from our results based on microsynteny related to miR167, miR167e in grape obviously remains in an orthologous relationship with group A (Figure 5). It should be noted that the whole-genome duplication events in grape are not definitively defined. Jaillon et al. [21] could not find evidence for a recent duplication in grape and they proposed that three ancestral genomes, resulted from an ancestral hexaploidization, contributed to the *Vitis *lineage. However, since many regions of the grape genome appear in triplicate, Velasco et al. [22] proposed that after the whole-genome duplication which was shared by all eudicots (and likely by the monocots), grape might have experienced a hybridization event shortly after its divergence from the lineage leading to *Populus *and Arabidopsis (see Fig. Eight of [22]). Obviously, the microsynteny of a single miR167 family cannot be applied with confidence to validate either of these two hypotheses, though the microsynteny and the Ks values suggest that, during grape speciation, the grape miR167e might come from outside of grape species. This seems to support the notion that genome duplication in grape likely had involved a hybridization event, as Velasco et al. [22] suggested.

**Table 2:** Estimation of the mean value of Ks for flanking genes around miR167s in Arabidopsis, *Populus*, grape and rice

miRNA		Ks	miRNA		Ks
ptc-miR167f	ptc-miR167g	0.29	ptc-miR167h	ptc-miR167a	1.95
ptc-miR167a	ptc-miR167c	0.33	ptc-miR167h	ptc-miR167g	1.75
ptc-miR167h	ptc-miR167e	0.31	ptc-miR167e	ptc-miR167g	2.13
ptc-miR167f	ptc-miR167h	1.96	ptc-miR167h	ptc-miR167c	1.9
ptc-miR167f	ptc-miR167c	1.66	ptc-miR167a	ptc-miR167e	2.6
ptc-miR167f	ptc-miR167a	1.79	ath-miR167a	ath-miR167b	1.83
ptc-miR167c	ptc-miR167g	2.27	vvi-miR167b	vvi-miR167e	1.32
ptc-miR167a	ptc-miR167g	2.38	vvi-miR167c	vvi-miR167e	1.33
ptc-miR167f	ptc-miR167e	1.75	vvi-miR167b	vvi-miR167c	2.03

The mean Ks value was calculated for each homologous pair of protein-coding genes between genomic fragments containing miR167s members, for which microsynteny have been detected.

##### Relationship and evolution of miR167s between monocots and eudicots

We used the miR167s in rice to search conserved regions in the genomes of Arabidopsis, *Populus *and grape to study the relationship of the miR167s between monocots and eudicots. With the inclusion of rice, it was difficult to classify A, B, C and D groups unambiguously to the groups in Arabidopsis, *Populus *and grape (Additional file 1: Supplemental Fig. S1-S3). Nevertheless, miR167e and miR167i appear to be the most ancient and are classified into group D; other miR167s in rice can only be roughly grouped as a composite "ABC" group. Although miR167d in Arabidopsis and miR167a in grape are likely ancient, weak microsynteny is detected with miR167e in rice and is thus grouped as group D. It has been well documented that the second whole-genome duplication event in rice occurred about 70 MYA, after the divergence of monocots and eudicots [23]. It has also been reported that colinear orthologs between monocots (rice) and the eudicots comprise only ~15% of rice genes distributed over about half of the genome [3]. In this study, it was also challenging to infer the contribution of this recent duplication to the miR167 duplication events, for that reason the orthologous correlation of miR167s in rice to group A, B or C in eudicots is unclear.

#### Verification of the phylogenetic tree of a protein-coding gene family

Since miRNA genes are very short in sequences by themselves to be used for constructing the phylogenetic trees, microsyntenies among/between their pre-miRNA genes are highly valuable in referring their evolutionary relationships. In using traditional phylogenetic tree to infer the member relationships, low bootstrap values (less than 60%) in some nodes are frequently observed, which suggests the low confidence in placing those gene members in those nodes. We used the XTH family in *Populus *as the test case for the reasons that it is not only an ancient gene family involved in cell wall biosynthesis (assumably appeared as early as vascular plants appeared), but also has undergone moderate gene expansion, therefore, enabling us to follow the evolutionary history.

**Figure 6:**
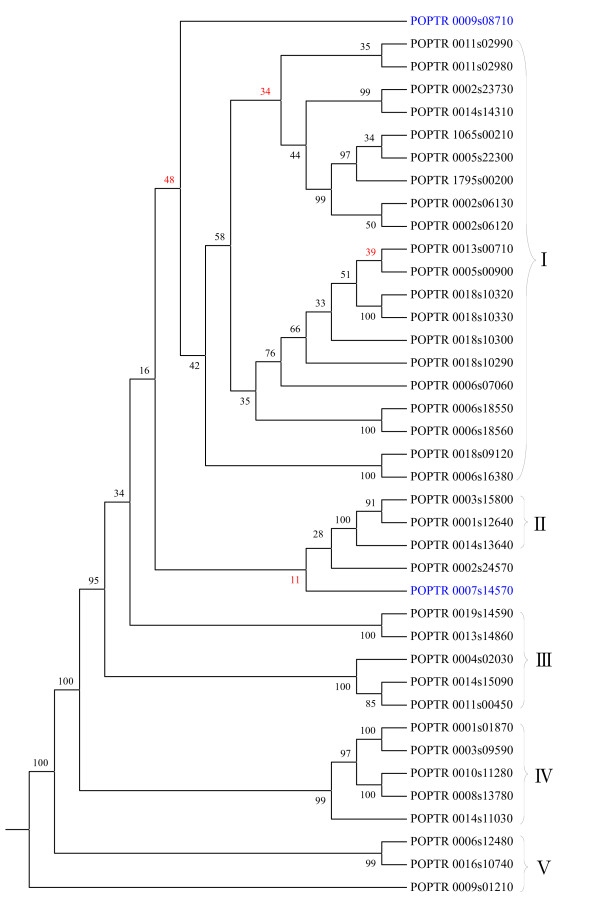
**Phylogenetic tree of 39 XTH genes from *Populus***. The tree was constructed using the maximum likelihood method, and the bootstrap values were showed. Branches with <60% bootstrap values in this tree and another NJ tree (Additional file 1: Supplemental Fig. S4) were marked with red. The genes of subfamily V are colored by blue.

**Figure 7:**
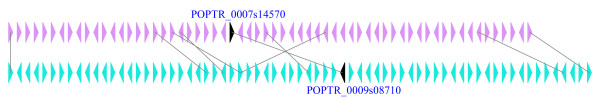
**Microsynteny related to two XTH genes, POPTR_0007s14570 and POPTR_0009s08710 in *Populus***. Triangles represent genes in a family and their flanking genes. The homologous genes on two fragments are connected by a gray line.

We firstly constructed the phylogenetic tree of 39 XTH genes in *Populus *using ML and NJ methods. In the process of building phylogenetic trees, the sequences were resampled to test their statistical significance using bootstrapping. To verify reliability of the phylogenetic trees representing phylogenetic predictions, the discrepancy in the two obtained phylogenetic trees and the branches with low bootstrap values (< 60%) were selected for searching their microsyntenies by MicroSyn. Initially, the phylogenetic analysis indicated that XTH gene family from *Populus *consists of four subfamilies of more closely related sequences, named here subfamily I (20 genes), II (3 genes), III (5 genes), IV (5 genes), and V (3 genes) (Figure 6, Additional file 1: Supplemental Fig. S4). In subfamily I, POPTR_0011s02980 has a bootstrap value 46 and 34 in NJ and ML trees, respectively, but POPTR_0011s02980 has a significant microsynteny with POPTR_0002s06130 in subfamily I; the bootstrap value between POPTR_0013s00710 and POPTR_0005s00900 is 39, and the microsynteny between them was detected (8e-31) (Additional file 1: Supplemental Table S1). It suggests that POPTR_0011s02980 and POPTR_0013s00710 belong to the subfamily I although with a low statistic support in bootstrap test. In two phylogenetic trees, POPTR_0007s14570 and POPTR_0009s08710 have different topology and low bootstrap values; the microsynteny between these two genes and other members in the five subfamilies were not detected, so these two genes cannot be assigned to any of the four groups. However, the microsynteny between these two genes is significant (1.7e-10), indicating that they are able to be classified into one subfamily, which has a week relationship with other four subfamilies, so we named it subfamily VI here (Figure 7). ML analysis shows that POPTR_0002s24570 is adjacent to subfamily II with a bootstrap value of 28, however, in the NJ tree, POPTR_0002s24570 is close to subfamily I or III. The MicroSyn analysis shows that POPTR_0002s24570 has a significant microsynteny with the POPTR_0006s18560 that is in subfamily I. It indicates that POPTR_0002s24570 belongs to subfamily I. In addition, POPTR_0014s11030, a member in subfamily IV, has significant microsyntenies with some genes in subfamily I, such as POPTR_0013s00710, POPTR_0018s10320, POPTR_0018s10330 and POPTR_0005s00900, suggesting that subfamily IV may have a closer relationship with subfamily I than with other subfamilies. For subfamily V, although the three members (POPTR 0006s12480, POPTR 0016s10740, and POPTR 0009s0121) are not in a monophyletic clade, the microsyntenies among them are significant, so we classified the three genes into a subfamily. These results demonstrated that MicroSyn can be applied to validating/correcting/adjusting the evolutionary relationships in poorly supported nodes in traditional phylogenetic trees.

## Discussion

### Comparison of MicroSyn with existing tools

The current tools related to synteny analysis include i-ADhoRe [14], DAGChainer [24], DiagHunter [25], FISH [26], Cinteny [27], Osfinder [28], OrthoClusterDB [29], SyntenyTracker [30], Satsuma [31], etc. In these tools, the target of analysis is conservative regions within or across species. If researchers are interested in the synteny relationship between regions that contain members in a gene family, they must extract all flanking regions of the genes of interest as the import data. A browser-based tool PLAZA Synteny plot http://bioinformatics.psb.ugent.be/plaza_v1/synteny/index reports the local gene organization for homologous genes within a family [15]. PLAZA Synteny plot presents some pre-computed information on the local gene organization of all homologs of that family. Users are allowed to choose three sets of window size (5, 10 or 15 genes), number of genes around the locus, to query the homologous relationship within a selected gene family. In comparison, MicroSyn is a stand-alone desktop software running in a GUI environment and is more flexible. User can use the family of interest that is not pre-computed in web-based databases, while other online tools are still limited by network latency and the load caused by simultaneous query of concurrent users. In MicroSyn, the window size or number of flanking genes around a member of a gene family is not fixed but can be set by the user, and other parameters of searching are also allowed to be defined by users. Before starting microsynteny detection, parameter settings are defined via user inputs. These inputs include 1) window size - the maximum number of adjacent genes that are allowed in the search on each gene list, 2) tandem gap size - the maximum number of genes allowed to be existed between two tandem homologous genes defined in the study, 3) homologous pairs-minimum number of homologous gene pairs in a detected microsynteny, and 4) expected threshold value - the maximum probability of a microsynteny estimate generated by chance. These parameters are used to customize the search results. In the two examples, the parameters were set as follows: window size of 100 genes, tandem gap value of 2, expected threshold value cut off of 0.01, and 3 homologous pairs to define a syntenic segment. For the window size, we suggest users to choose a value > 30 genes, because a too small window size will decrease the sensitivity.

### Application in two examples

For miR167 we only considered the conserved synteny of miR167s intra- and inter-species without using the gene sequences themselves to successfully infer the duplication events of this gene family in plants. This was particularly successful in eudicots, where the microsynteny of miR167s can not only be used to infer the relationships within one species (relationship and evolution resulted from genome or gene duplication), but also be used to infer the orthologous relationships between and among the species (Arabidopsis, *Populus *and grape). A less definite inference between monocots and eudicots using microsynteny and colinearity was reasonable and possibly due to the far divergence of monocots and eudicots [3]. However, the user-friendly MicroSyn software has been proven to be a new means to study the evolution of gene family among moderately diverged species. We also applied MicroSyn to validate/adjust the evolutionary relationship in the questionable nodes (with low bootstrap values) in traditional phylogenetic trees, such as ML or NJ trees, of a protein-coding gene family, the XTH gene family. Based on the phylogenetic tree of the XTH family constructed by traditional methods using their amino acid sequence homologies, the microsynteny between each pair of members can be easily obtained by using the MicroSyn software. In several poorly supported branches/nodes, the microsynteny can be useful to further check or modify the phylogenetic tree.

### Limitations of MicroSyn

MicroSyn is suitable for the analysis of a small or medium size of gene family. If a large set of sequences need to be analyzed, it is better to decrease the window size. Choosing a smaller window size will cause smaller region to be searched for the colinearity between genomic segments. The larger size of flanking region, the greater chance that a synteny will be found. In essence, when a larger widow size is set, the sensitivity is increased while the probability of Random-access memory (RAM) overflows is also increased. By modulating the window size, users can find the best balance of precision and performance that best suits his or her needs. In this study, the two examples were performed on a 2.8 GHz Intel Pentium 4 CPU and 4G RAM based PC with Windows XP operating system. For the XTH family, the window size of flanking genes is set as 100 and the process of detecting cost 7 minutes.

Microsynteny between two members of a gene family is calculated from their flanking genes. If the flanking regions contain assembly errors, gaps or annotation errors, the microsynteny should be artificial. So users should be cautious about it.

## Conclusions

In this study, we took advantages of newly available whole genome resources, and developed a program, MicroSyn, a semi-automated tool, to provide an additional valuable means to address the problem intrinsic in the computational methods and sequence qualities themselves in gene family analysis. MicroSyn is particularly useful for identification of conserved synteny among regions surrounding genes in a gene family. This tool can facilitate research related to defining the genome evolution and gene duplication events within a gene family that have led to the extant gene catalog. We demonstrated the utility of this software in the case study of miR167, a miRNA gene family whose evolutionary relationship cannot be inferred based on the traditional phylogenetic tree analysis due to short conserved sequences of these miRNAs. We also demonstrated that MicroSyn can be used to validate or correct the evolutionary relationships in poorly supported nodes in traditional phylogenetic trees.

We plan to continue improving the facility of operation, based on user-feedbacks. In the future, more sophisticated modules, such as automatic tree reconstruction based on the microsynteny information between members of a gene family using some more sophisticated methods. MicroSyn is now implemented in C# on Windows platform. We also plan to produce Mac or Linux versions, since most genomic scientists use these operating systems.

## Availability and requirements

**Project name**: MicroSyn

**Project home page**: http://fcsb.njau.edu.cn/microsyn

**Operating system(s)**: Windows

**Programming language**: C#

**Requirements**: .net framework on Windows

## Abbreviations

WGD: whole genome duplication; GUI: graphical user interface; miRNAs: microRNAs; XTH: Xyloglucan Endotransglycosylase/Hydrolase; CDS: coding sequence; ML: maximum likelihood; NJ: neighbor jointing; MYA: million years ago; Ks: synonymous substitution; RAM: Random-access memory.

## Authors' contributions

BC and ZMC conceived the initial concept, discussed with XHY and GAT, BC carried out the research and BC, XHY, GAT and ZMC wrote the paper. All authors read and approved the final manuscript.

## Supplementary Material

Additional file 1 **Figure S1 - Microsynteny related to miR167 families between Arabidopsis and rice**. Figure S2 - Microsynteny related to miR167 families between *Populus *and rice. Figure S3 - Microsynteny related to miR167 families between grape and rice. Figure S4 - Phylogenetic tree of 39 XTH genes from *Populus*. Table S1 - Microsynteny of XTH genes in *Populus*. Click here for file
