# Supplementary material for: MicroSyn: A user friendly tool for detection of microsynteny in a gene family
Source: BMC Bioinformatics. 2011 Mar 18;12:79. doi: 10.1186/1471-2105-12-79 (PMC3072343; doi:10.1186/1471-2105-12-79)
Supplement: Additional file 1 — Figure S1 - Microsynteny related to miR167 families between Arabidopsis and rice. Figure S2 - Microsynteny related to miR167 families between Populus and rice. Figure S3 - Microsynteny related to miR167 families between grape and rice. Figure S4 - Phylogenetic tree of 39 XTH genes from Populus. Table S1 - Microsynteny of XTH genes in Populus. [file 1471-2105-12-79-S1.PDF]

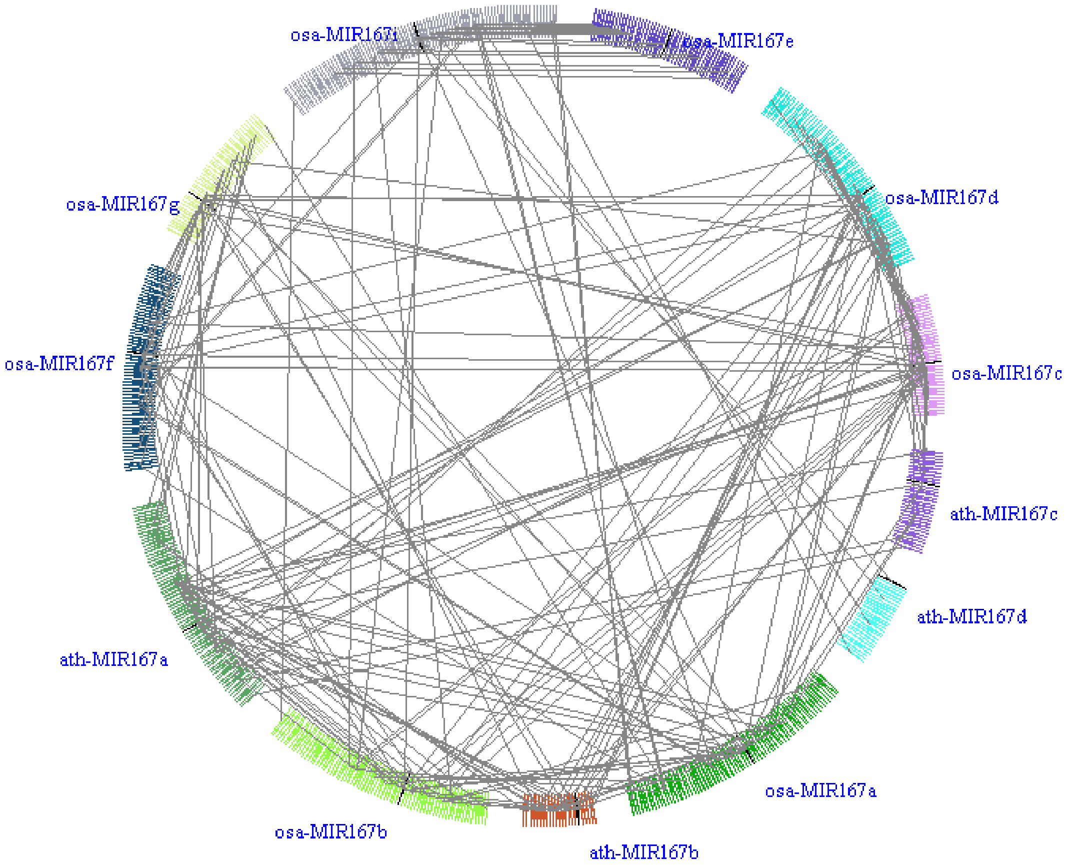

Figure S1 - Microsynteny related to miR167 families between Arabidopsis and rice

Use: ath for Arabidopsis, osa for rice.

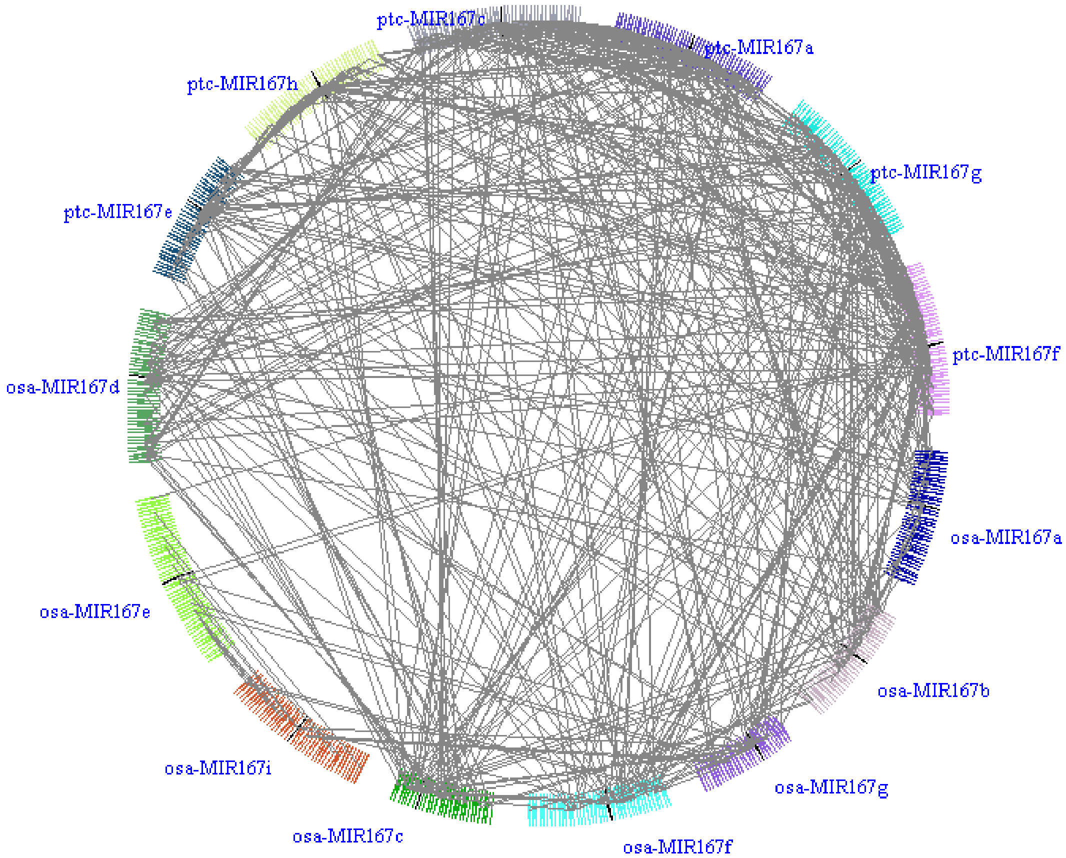

Figure S2 - Microsynteny related to miR167 families between *Populus* and rice

Use: ptc for *Populus*, osa for rice.

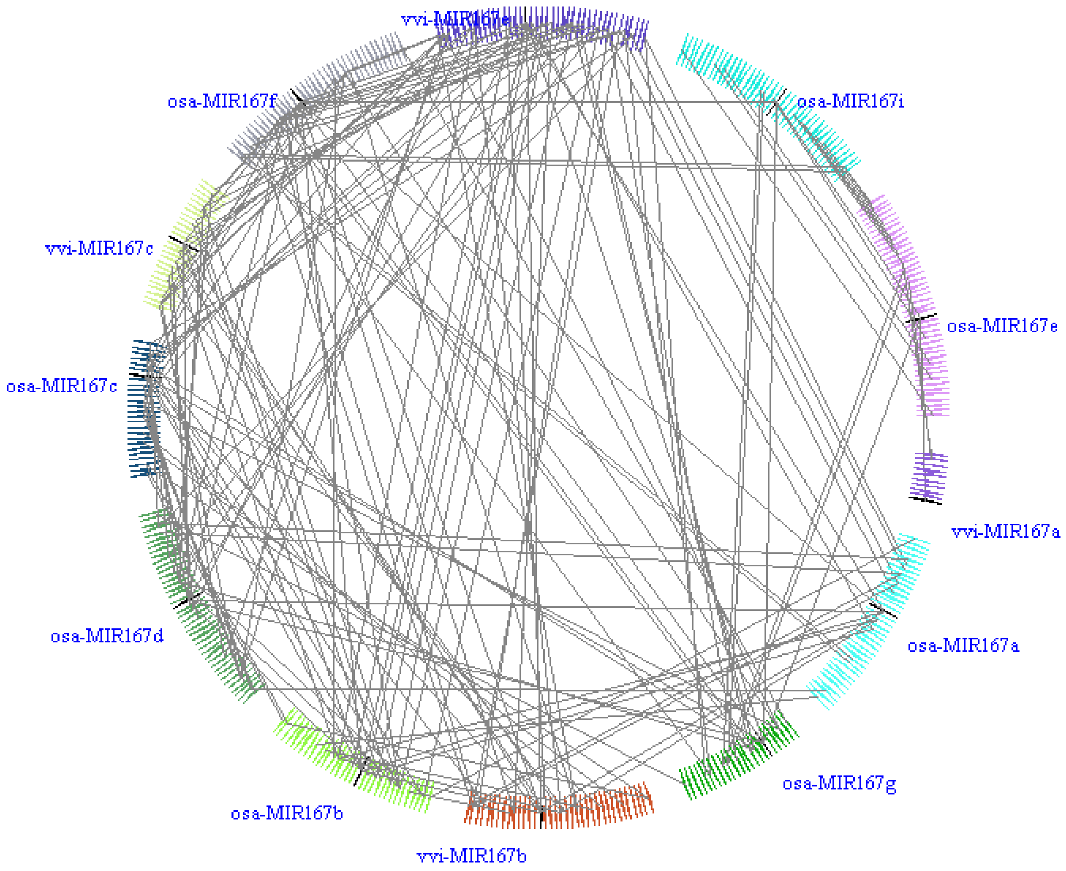

Figure S3 - Microsynteny related to miR167 families between grape and rice

Use: vvi for grape, osa for rice.

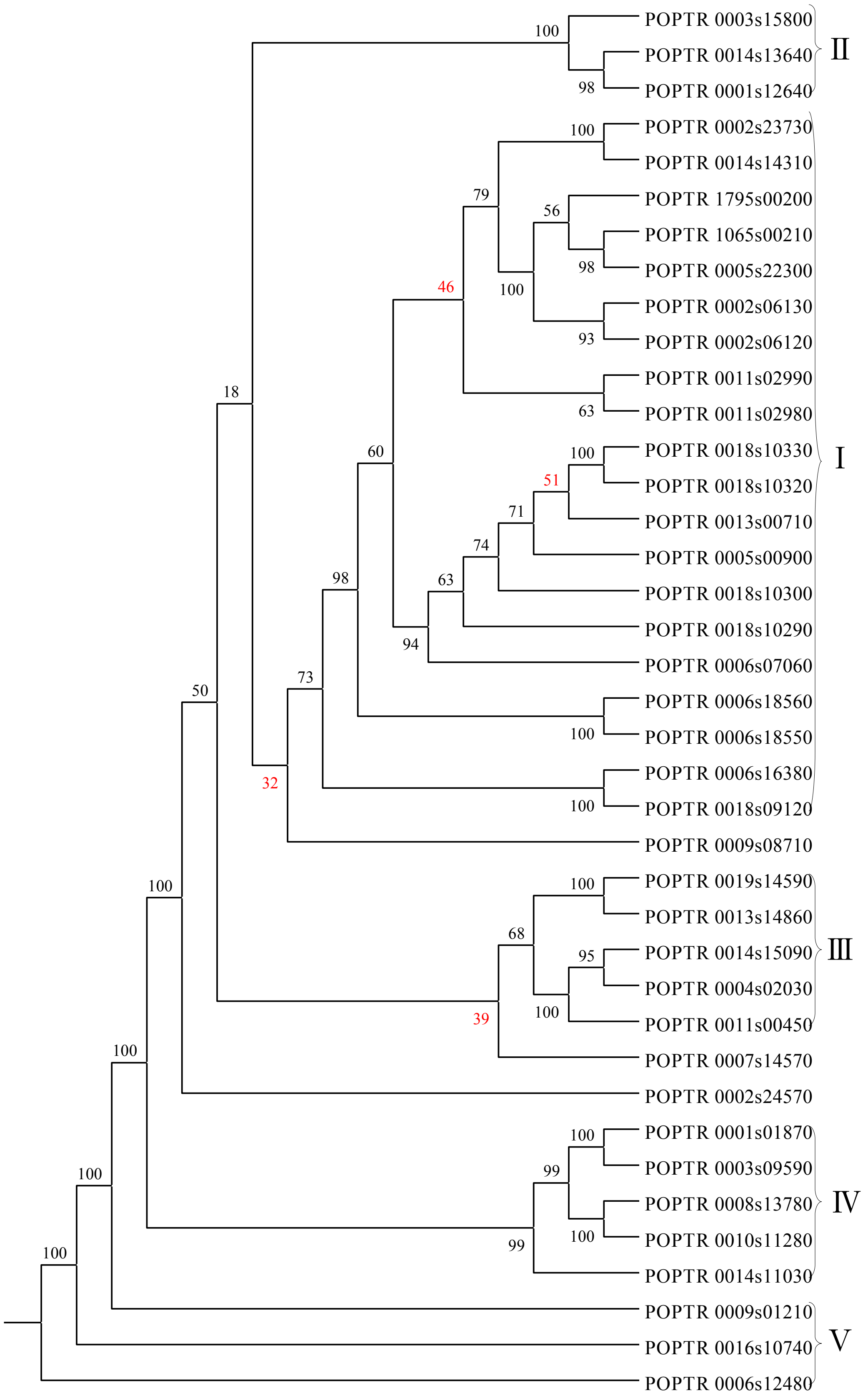

Figure S4 - Phylogenetic tree of 39 XTH genes from *Populus*

The tree was constructed using the neighbor joining method, and the bootstrap values were showed. Branches with <60% bootstrap value both in this tree and another ML tree (Fig. 6) were marked with red.

Table S1 – Microsynteny of XTH genes in *Populus*

Genomic fragments		Count of homologies	Expected value
POPTFR_0001s12640	POPTR_0003s15800	59	5.13E-104
POPTR_0002s06120	POPTR_0005s22300	59	1.64E-103
POPTR_0010s11280	POPTR_0008s13780	55	4.55E-95
POPTR_0001s01870	POPTR_0003s09590	50	1.42E-88
POPTR_0018s10290	POPTR_0006s18560	38	1.02E-55
POPTR_0013s14860	POPTR_0019s14590	30	7.70E-46
POPTR_0018s10290	POPTR_0006s18550	29	1.86E-43
POPTR_0013s00710	POPTR_0014s11030	21	6.17E-17
POPTR_0013s00710	POPTR_0005s00900	20	7.99E-31
POPTR_0016s10740	POPTR_0006s12480	17	4.06E-24
POPTR_0011s00450	POPTR_0004s02030	12	5.27E-20
POPTR_0011s02980	POPTR_0011s02990	11	3.27E-09
POPTR_0007s14570	POPTR_0009s08710	10	1.68E-10
POPTR_0014s14310	POPTR_0002s23730	9	6.35E-16
POPTR_0002s24570	POPTR_0006s18560	9	5.64E-12
POPTR_0006s12480	POPTR_0009s01210	9	7.78E-11
POPTR_0006s18550	POPTR_0006s18560	9	5.26E-11
POPTR_0018s09120	POPTR_0006s16380	8	1.04E-10
POPTR_0018s10290	POPTR_0002s06130	8	8.68E-07
POPTR_0001s12640	POPTR_0011s02990	6	1.03E-06
POPTR_0011s00450	POPTR_0005s00900	6	0.000416228
POPTR_0011s02980	POPTR_0002s06130	6	1.26E-08
POPTR_0018s10290	POPTR_0002s06120	6	0.000416545
POPTR_0018s10290	POPTR_0005s22300	6	0.000870947
POPTR_0011s00450	POPTR_0011s02990	5	2.42E-06
POPTR_0011s02980	POPTR_0014s14310	5	0.00039786
POPTR_0011s02980	POPTR_0002s06120	5	3.10E-06
POPTR_0011s02980	POPTR_0005s22300	5	9.09E-07
POPTR_0014s11030	POPTR_0018s10320	5	1.53E-06
POPTR_0014s11030	POPTR_0018s10330	5	4.03E-06
POPTR_0014s11030	POPTR_0005s00900	5	0.000373364
POPTR_0014s15090	POPTR_0006s18560	5	1.06E-05
POPTR_0016s10740	POPTR_0009s01210	5	3.35E-05
POPTR_0005s00900	POPTR_0005s22300	5	0.002313719
POPTR_0011s00450	POPTR_0008s13780	4	0.006479493
POPTR_0014s11030	POPTR_0014s14310	4	0.002391109
POPTR_0014s11030	POPTR_0018s10300	4	6.10E-05
POPTR_0014s11030	POPTR_0006s18560	4	8.07E-05
POPTR_0014s14310	POPTR_0004s02030	4	0.004683694
POPTR_0014s15090	POPTR_0002s06130	4	0.000122776
POPTR_0018s10290	POPTR_0018s10320	4	0.001051844
POPTR_0018s10290	POPTR_0018s10330	4	0.002489619
POPTR_0002s06120	POPTR_0006s18560	4	8.11E-05
POPTR_0002s23730	POPTR_0008s13780	4	0.005306954

POPTR_0002s24570	POPTR_0005s22300	4	0.008327625
POPTR_0001s01870	POPTR_0010s11280	3	0.006459804
POPTR_0001s01870	POPTR_0008s13780	3	0.005862955
POPTR_0010s11280	POPTR_0003s09590	3	0.005604381
POPTR_0013s00710	POPTR_0013s14860	3	0.002963791
POPTR_0013s14860	POPTR_0014s11030	3	0.007446736
POPTR_0013s14860	POPTR_0005s00900	3	0.008876535
POPTR_0014s14310	POPTR_0003s09590	3	0.003113696
POPTR_0019s14590	POPTR_0003s09590	3	0.003760824
POPTR_0003s09590	POPTR_0008s13780	3	0.002932297

Microsyntenies of 39 XTH genes in *Populus* were analyzed using MicroSyn, based on the following parameters: tandem gap size of 2, homologous pairs of 3 and expected threshold value of 0.01 to define a significant microsynteny between each pair of members in gene family. The count of flanking genes around each XTH gene was set as 100.
